# Supplementary material for: Yap haploinsufficiency leads to Müller cell dysfunction and late-onset cone dystrophy
Source: Cell Death Dis. 2020 Aug 14;11(8):631. doi: 10.1038/s41419-020-02860-9 (PMC7429854; doi:10.1038/s41419-020-02860-9)
Supplement: Supplementary file 1 — Supplementary Figure Legends [file 41419_2020_2860_MOESM1_ESM.docx]

SUPPLEMENTARY FIGURE LEGENDS

**Supplementary Figure S1. Absence of retinal progenitor proliferation from P11 *Yap^+/-^* mice.** (**A**) Timeline diagram of the experimental procedure used in B. Wild-type (Control) or *Yap^+/-^* mice were injected with EdU at P11 and the presence of EdU-positive cells was assessed 24 hours later (P12). (**B**) Retinal sections labelled for EdU (red) and DAPI counterstained (blue). Ventral areas are enlarged in the bottom panels. INL: inner nuclear layer; ONL: outer nuclear layer; GCL: ganglion cell layer. Scale bar: 200 µm and 50 µm (enlarged panels).

**Supplementary Figure S2. Correct cell fate of retinal progenitors with delayed cell cycle exit in *Yap^+/-^* mice** (**A**) Timeline diagram of the birthdating experimental procedure used in B. Wild-type (Control) or *Yap^+/-^* mice were injected with EdU at P6 and the fate of EdU-positive cells was analysed in 2-month-old mice. (**B**) Retinal sections labelled for EdU (red) and DAPI counterstained (blue). The delineated areas are enlarged in the insets. The asterisk marks the location of a dysplastic region. (**C**) Retinal sections labelled for EdU (red) and stained for the indicated markers (SOX9, CHX10, Recoverin, green) and DAPI counterstained (blue). Arrows point to double labelled cells. (**D, E**) Quantification of double labelled cells among EdU-positive cells per field (400 µm x 400 µm) in the central (D) or peripheral (E) retinal region. Mean values ± SEM from 3 retinas per condition are shown. INL: inner nuclear layer; ONL: outer nuclear layer; GCL: ganglion cell layer. Statistics: Mann-Whitney test, ns: non-significant. Scale bar: 200 µm (B), 50 µm (C).

**Supplementary Figure S3. One allele deletion of *Yap* does not alter eye size but generate retinal dysplasia**. (**A**) Enucleated eyes from wild type (Control) and *Yap*^+/-^ 12-month-old mice. (**B**) H&E staining of retinal sections in 4 and 12-month-old wild type (Control) and *Yap^+/-^* mice. Red asterisks indicate the position of dysplastic regions. Area delineated with dashed black lines are enlarged in panels on the right showing finger-like protrusions of *Yap^+/-^* retinal layers toward the RPE. (**C**) Number of retinas with a dorsal, central or ventral dysplasia in *Yap*^+/-^ retinas expressed in percentage of the total number of retinas with a dysplasia (n=96 retinas from stage p21 to 12 month-old). (**D**) Incidence of retinal dysplasia in control and *Yap*^+/-^ mice at different stages. INL: inner nuclear layer; ONL: outer nuclear layer; GCL: ganglion cell layer. Scale bar: 200 µm and 50 µm (enlarged panels).

**Supplementary Figure S4.** **Normal appearance of the RPE and normal retinal thickness in *Yap^+/-^* mice**. (**A**) RPE flat mounts from 12-month-old wild type (control) and *Yap^+/-^* mice immunostained for Phalloidin (red) and RPE65 (green). Nuclei are DAPI counterstained (blue). (**B**) Analysis of RPE65 and α-tubulin (α-Tub) level of expression in 1 or 12-month-old mice. (**C**) RPE sections from 12-month-old mice immunostained for OTX2 (red) and Ezrin (green). Nuclei are DAPI counterstained (blue). RPE: retinal pigment epithelium. **(D)** H&E staining of retinal sections from 12-month-old wild type (Control) and *Yap^+/-^* mice. A region away from a dysplastic region is shown. Histogram represents the measurement of the outer and inner nuclear layer thickness. Mean values ± SEM from three independent retinas are shown. INL: inner nuclear layer; ONL: outer nuclear layer; GCL: ganglion cell layer. Mean ± SEM from 7 retinas per condition are shown. Statistics: Mann-Whitney test, ns: non-significant. Scale bar: 20 µm and 100 µm (enlarged panels) (A), 10 µm (C), 50 µm (D).

**Supplementary Figure S5.** **Altered cone-driven vision in *Yap*^+/-^ adult mice.** (**A**) Representative ERG intensity series for scotopic (dark-adapted) and photopic (light-adapted) responses in 12-month-old wild-type (Control) and *Yap*^+/-^ mice. (**B**) Quantitative evaluation of the scotopic and photopic a- and b-waves maximum amplitude data from 4- and 8-month-old wild-type (black) or *Yap*^+/-^ (red) mice. Mean ± SEM intensity response curves are averaged from: 8 controls and 7 *Yap*^+/-^ biological replicates of 4- month-old mice; 9 controls and 8 *Yap*^+/-^ biological replicates of 8-month-old mice.

**Supplementary Figure S6**. **Altered architecture of the** **photoreceptor ribbon synapse in 12-month-old *Yap*^+/-^ adult mice**. 12-month-old retinal sections immunostained for PKC-α (green) and RIBEYE (red). Delineated areas (dashed lines) are enlarged in the right panels. Nuclei are DAPI counterstained (blue). An arrow indicates a ribbon with a normal horseshoe shape, facing PKC-α labelled rod-bipolar cell post-synaptic terminals in *Yap^+/-^* retinas. The scatter plot with bars indicates the number of labelled RIBEYE positive ribbons per field (150 µm x 150 µm). Values are expressed as the mean ± SEM from 3 retinas per condition. ONL: outer nuclear layer, INL: inner nuclear layer, GCL: ganglion cell layer. Statistics: Mann-Whitney test, ns: non-significant. Scale bar: 20 µm and 50 µm (enlarged panels).

**Supplementary Figure S7.** **Decreased expression of cone markers in 12-month-old *Yap*^+/-^ mice retinas. (A)** Retinal flat-mounts from 12-month-old wild type (control) and *Yap*^+/-^ mice, immunostained for cone makers (PNA, S-Opsin and M-Opsin) in the ventral part of the retina. Histograms indicate the number of labelled cells per field (400 µm x 400 µm). Values are expressed as the mean ± SEM from 6 retinas per condition. **(B)** 12-month-old mouse retinal sections immunostained for PNA (red) and Cone Arrestin (green). Nuclei are DAPI counterstained (blue). Scatter plots with bars represent the number of Cone Arrestin labelled cells per field (150 µm x 150 µm). Values are expressed as the mean ± SEM from 3 retinas per condition. ONL: outer nuclear layer, INL: inner nuclear layer, GCL: ganglion cell layer. Scale bar: 50 µm (A), 20 µm (B). Statistics: Mann-Whitney test, *p≤ 0.05, **p≤ 0.01.

**Supplementary Figure S8. Normal expression of cone markers in 1- and 4-month-old *Yap*^+/-^ mice**. **(A)** 1-month-old retinal sections immunostained for cone markers (PNA, S-opsin, M-opsin). Scatter plots with bars represent the quantification of labelled cells per field (300 µm x 300 µm). Central, mid-dorsal and ventral regions of retinal sections are shown. Mean values ± SEM from 3 retinas per condition are shown. INL: inner nuclear layer; ONL: outer nuclear layer; GCL: ganglion cell layer. Statistics: Mann-Whitney test, ns: non-significant. **(B)** 4-month-old retinal flat-mounts immunostained for cone markers (PNA, S-opsin, M-opsin). Scatter plots with bars represent the quantification of labelled cells per field (400 µm x 400 µm). Mean values ± SEM from 3 retinas per condition are shown. Statistics: Mann-Whitney test, ns: non-significant. Scale bare : 20 µm (A), 50 µm (B).

**Supplementary Figure S9. Normal expression of Rhodopsin and normal number of Müller cells in *Yap*^+/-^ adult mice**. 12-month-old retinal sections immunostaining for Rhodopsin (Rho, red) **(A)** or for SOX9 (green) **(B)**. Nuclei are DAPI counterstained (blue). Scatter plots with bars represents the quantification of labelled cells per field (1200 µm x 1200 µm). Mean values ± SEM from 3 retinas per condition are shown. GCL: ganglion cell layer, INL: inner nuclear layer; ONL: outer nuclear layer; OS: outer segment. Statistics: Mann-Whitney test, ns: non-significant. Scale bar: 50 µm.

**Supplementary Figure S10. Alteration of intermediate retinal vascular plexus in *Yap*^+/-^ adult mice.** (**A**) Schematic representation of the three-retinal plexi: deep plexus, intermediate plexus and superficial plexus (red) in the mouse retina. This figure was created with some schemas adapted from Servier Medical Art by Servier (licensed under a Creative Commons Attribution 3.0 Unported License). (**B**) 8 and 12-old-month retinal flat-mounts stained for endothelial cells with isolectin B_4_ (red) in the superficial, intermediate, and deep plexi (n=3 mice per condition). INL: inner nuclear layer; ONL: outer nuclear layer; GCL: ganglion cell layer; RPE: retinal pigment epithelium. Scale bar: 100 µm.

**Supplementary Figure S11. No blood leakage in *Yap*^+/-^ adult mice.** Fluorescein angiography in 12-month-old mouse retina showing no signs of vascular leakage approximately 5 min after intra-peritoneal injection of sodium fluorescein. Scale bar: 200 µm.

**Supplementary Table S1: List of primers.**

**Supplementary Table S2: List of antibodies.** IHC: immunohistochemistry, WB: western blot.
