# Supplementary material for: Yap haploinsufficiency leads to Müller cell dysfunction and late-onset cone dystrophy
Source: Cell Death Dis. 2020 Aug 14;11(8):631. doi: 10.1038/s41419-020-02860-9 (PMC7429854; doi:10.1038/s41419-020-02860-9)
Supplement: Supplementary file 13 — Table S1 [file 41419_2020_2860_MOESM13_ESM.docx]

**Table S1**

| Gene | Forward primer (5’-3’) | Reverse primer (5’-3’) |
| --- | --- | --- |
| *Ctgf (Connective tissue growth factor)* | GCCCTAGCTGCCTACCGACT | AGAACAGGCGCTCCACTCTG |
| *Cyr61 (Cysteine-rich angiogenic inducer 61)* | TGAAGAGGCTTCCTGTCTTTGG | CGGCACTCTGGGTTGTCATT |
| *Egfr (Epidermal growth factor receptor)* | TGGCATCATGGGAGAGAACA | GTACAGTTGGCGTGGCATAG |
| *Erbb2 (Erb-B2 tyrosine kinase receptor 2)* | AACTGCAGTCAGTTCCTCCG | GTGCTTGCCCCTCACATACT |
| *Erbb3 (Erb-B2 tyrosine kinase receptor 3)* | AGTCCGGGAGATTACAGGCT | CGATGGTCGTCAGGTTGGAA |
| *Erbb4 (Erb-B2 tyrosine kinase receptor 4)* | CCTTGCCATCCAAACTGCAC | GGTAAAGTGGAATGGCCCGT |
| *Gfap (Glial fibrillary acidic protein)* | CCAGTTACCAGGAGGCACTTG | CGATGTCCAGGGCTAGCTTAA |
| *Hbegf (Heparin binding epidermal growth factor)* | GCATCCAAAGTGATCGCTGC | GACAACACTGCGGCCAGAAA |
| *Nrg1 (Neuregulin 1)* | GAGTGCAGACCCATCTCTCG | CCAGGGCTTCTCCCATCTTC |
| *Rax-CreERT2 (genotyping)* | TTCCCGCAGAACCTGAAGAT | CCCCAGAAATGCCAGATTAC |
| *Rps26 (Mitochondrial ribosomal S26 protein)* | CCCAAGGATAAGGCCATCAAG | AAGCACGTAGGCGTCGAAGA |
| *Srp72 (Signal Recognition Particle 72)* | CACCCAGCAGACAGACAAACTG | GCACTCATGGTAGCGTTCCA |
| *Taz/wwtr1 (WW domain containing transcription regulator 1)* | AGACCCCAGGAAGGTGATGAA | TGTTGGTGATTCATTGCGAGAT |
| *Tead1 (TEA domain family member 1)* | TCTGGGCGGACTTAAACTGC | GAACCTCGCATACTCCGTCTCT |
| *Tead2 (TEA domain family member 2)* | CGGAAGCCTGGTTATTGAGC | TTCCAGAAGAGCAAGGAGGG |
| *Yap (Yes-associated-protein 1)* | TCCTGATGGATGGGAGCAAG | CTCTGGTTCATGGCAAAACGA |
