# Supplementary material for: Yap haploinsufficiency leads to Müller cell dysfunction and late-onset cone dystrophy
Source: Cell Death Dis. 2020 Aug 14;11(8):631. doi: 10.1038/s41419-020-02860-9 (PMC7429854; doi:10.1038/s41419-020-02860-9)
Supplement: Supplementary file 14 — Table S2 [file 41419_2020_2860_MOESM14_ESM.docx]

**Supplementary Table S2**

| **Antigene** | **Host** | **Supplier** | **Reference** | | **Dilution (IHC)** | **Dilution**  **(WB)** |  |
| --- | --- | --- | --- | --- | --- | --- | --- |
| **Primary antibodies** | | | | | | | |
| **AKT** | mouse | SIGMA | | P2482 |  | 1:1,000 |  |
| **Alpha-tubulin** | mouse | SIGMA | | T5168 |  | 1:20,000 |  |
| **AQP4** | rabbit | Alomone labs | | AQP4-004 | 1:300 | 1:3000 |  |
| **CHX10** | mouse | Santa cruz | | SC365519 | 1:250 |  |  |
| **CRALPB** | mouse | abcam | | ab15051 | 1:50 | 1:500 |  |
| **CtBP2/ribeye** | mouse | BD Bioscience | | BD612044 | 1:50 |  |  |
| **Cycline D1** | rabbit | abcam | | ab134175 | 1:100 |  |  |
| **ERK1/2 (p44/42 MAPK)** | rabbit | Cell Signaling | | 9102 |  | 1:1,000 |  |
| **EZRIN** | mouse | Neomarkers | | MS-661PABX | 1 :1,000 |  |  |
| **GFAP** | rabbit | Dako | | Z0334 | 1:500 |  |  |
| **GFAP** | goat | Abcam | | ab53554 |  | 1:1,500 |  |
| **Glutamine synthetase** | mouse | Abcam | | ab64613 | 1:1,500 | 1:10,000 |  |
| **Isolectin gs-ib4 alexa 594** | G.simplicifolia | Thermo Fisher Scientific | | 121413 | 1:1,500 |  |  |
| **Kir4.1** | rabbit | Alomone labs | | APC-035 | 1:300 | 1:500 |  |
| **Lectin PNA alexa 568** | peanut | Thermo Fisher Scientific | | L32458 | 1:500 |  |  |
| **OTX2** | rabbit | abcam | | ab114138 | 1:200 |  |  |
| **P-AKT (Ser473)** | rabbit | Cell Signaling | | 9271 |  | 1:1,000 |  |
| **PCNA** | mouse | Dako | | Mo879 | 1:500 |  |  |
| **P-ERK1/2 (Thr202/Tyr204)** | rabbit | Cell Signaling | | 4370 |  | 1:1,000 |  |
| **Phalloidin alexa 568** | A.phalloides | Thermo Fisher Scientific | | A12380 | 1:50 |  |  |
| **PKC alpha** | rabbit | Sigma | | P4334 | 1:400 |  |  |
| **P-Stat3 (Ty705)** | mouse | Cell Signalling | | 9138S | 1:1000 |  |  |
| **Recoverin** | rabbit | millipore | | AB5585 | 1:100 |  |  |
| **Rhodopsin** | mouse | EMD Millipore | | MAB5316 | 1:2,000 |  |  |
| **M-opsin** | rabbit | EMD Millipore | | AB5407 | 1:1000 |  |  |
| **Stat3** | mouse | Cell Signalling | | 9139S | 1:1000 |  |  |
| **S-opsin** | rabbit | EMD Millipore | | AB5407 | 1:500 |  |  |
| **Sox9** | rabbit | EMD Millipore | | AB5535 | 1:300 |  |  |
| **RPE65** | mouse | Thermo fisher Scientific | | SC53483 | 1:500 | 1:1,000 |  |
| **TAZ**  **TEAD-1** | Rabbit  Rabbit | Abcam  Abcam | | Ab110239  Ab133533 |  | 1:1,000  1:500 |  |
| **YAP** | mouse | Abcam | | ab56701 | 1:50 | 1:1,000 |  |
| **Secondary antibodies** | | | | | | | |
| **Alexa 488 anti-mouse IgG1** | goat | Thermo Fisher Scientific | | A21121 | 1:200 |  |  |
| **Alexa 555 anti-mouse IgG1** | goat | Thermo Fisher Scientific | | A21127 | 1:200 |  |  |
| **Alexa 488 anti-mouse IgG2a** | goat | Thermo Fisher Scientific | | A21141 | 1:200 |  |  |
| **Alexa 647 anti-rabbit** | goat | Thermo Fisher Scientific | | A21244 | 1:200 |  |  |
| **HRP anti-mouse IgG** | goat | Sigma-Aldrich | | A4416 |  | 1:5,000 |  |
| **HRP anti-rabbit IgG** | donkey | GE Health | | NA934V |  | 1:5,000 |  |
| **HRP anti-goat IgG** | rabbit | Sigma-Aldrich | | A5520 |  | 1:5,000 |  |
